# A novel automatic flow method with direct-injection photometric detector for determination of dissolved reactive phosphorus in wastewater and freshwater samples

**DOI:** 10.1007/s10661-018-6511-z

**Published:** 2018-02-12

**Authors:** Stanislawa Koronkiewicz, Mihaela Trifescu, Lech Smoczynski, Harsha Ratnaweera, Slawomir Kalinowski

**Affiliations:** 1 Department of Chemistry, University of Warmia and Mazury, 10-957 Olsztyn, Poland; 2 Faculty of Science and Technology, Norwegian University of Life Sciences, Aas, Norway

**Keywords:** Direct-injection detector, Light-emitting diode, Multi-pumping flow system, Phosphate determination, Wastewater

## Abstract

The novel automatic flow system, direct-injection detector (DID) integrated with multi-pumping flow system (MPFS), dedicated for the photometric determination of orthophosphates in wastewater and freshwater samples is for the first time described. All reagents and the sample were injected simultaneously, in counter-current into the reaction-detection chamber by the system of specially selected for this purpose solenoid micro-pumps. The micro-pumps provided good precision and accuracy of the injected volumes. For the determination of orthophosphates, the molybdenum blue method was employed. The developed method can be used to detect orthophosphate in the range 0.1–12 mg L^−1^, with the repeatability (RSD) about 2.2% at 4 mg L^−1^ and a very high injection throughput of 120 injections h^−1^. It was possible to achieve a very small consumption of reagents (10 μL of ammonium molybdate and 10 μL of ascorbic acid) and sample (20 μL). The volume of generated waste was only 440 μL per analysis. The method has been successfully applied, giving a good accuracy, to determination of orthophosphates in complex matrix samples: treated wastewater, lake water and reference sample of groundwater. The developed system is compact, small in both size and weight, requires 12 V in supply voltage, which are desirable for truly portable equipment used in routine analysis. The simplicity of the system should result in its greater long-time reliability comparing to other flow methods previously described.

## Introduction

Phosphorus is an essential nutrient for plants, animals and humans. It is beneficial to many biological processes in the environment. However, too much phosphate in water can contribute to eutrophication. Therefore, phosphorus removal is essential in wastewater treatment and should be monitored.

Knowledge of the concentration of phosphorus, along with related physico-chemical data, can assist in controlling the wastewater treatment plant (WWTP) so that efficient removal is achieved. One of the potential applications of online analyses is controlled dosing of coagulants (e.g. iron and aluminium salts) in chemical sewage treatment plants (Benson et al. 1996). An online phosphorus analyser would contribute to the most cost-efficient work of a treatment plant and reduce the phosphorus content to an acceptable level in terms of both the quality of receiving water and legal requirements.

Most chemical parameters can be analysed online today. Phosphate is one of the few parameters which still cannot be measured in true real time, as only wet chemistry-based flow autoanalysers are commercially available. Some wastewater treatment plants use nowadays such systems, but, to our knowledge, the biggest weakness is that they can at best analyse four to six samples per hour.

The most bioavailable form of phosphorus in aquatic ecosystem, orthophosphate, is usually measured as dissolved reactive phosphorus (DRP), which is filterable by the 0.45-μm membrane (Benson et al. 1996; Closceri et al. 1998). Most methods of DRP determination in natural water and wastewater are based on the spectrophotometric detection of complex phosphomolybdenum blue. This is an official, standard method recommended by the American Public Health Association (Closceri et al. 1998), which is also accepted by most national standards. The molybdenum blue reaction occurs in two stages: the first involves reaction of phosphate with acidified molybdate, producing 12-molybdophosphoric acid (McKelvie et al. 1995; Worsfold et al. 2016):

1 $$ {{\mathrm{PO}}_4}^{3-}\kern0.5em +\kern0.5em 12{{\mathrm{Mo}\mathrm{O}}_4}^{2-}+27{\mathrm{H}}^{+}\to {\mathrm{H}}_3{\mathrm{PO}}_4{\left({\mathrm{Mo}}_3\right)}_{12}+12{\mathrm{H}}_2\mathrm{O} $$

In the second stage, the produced heteropolyacid is reduced to form deeply blue-coloured phosphomolybdenum blue:

2 $$ {\mathrm{H}}_3{\mathrm{PMo}}_{12}{\mathrm{O}}_{40}+\mathrm{reductant}\to {\left[{\mathrm{H}}_4{\mathrm{PMo}}_8{\mathrm{Mo}}_4{\mathrm{O}}_{40}\right]}^{3-} $$

A variety of reductants (e.g. ascorbic acid, tin(II) chloride, hydrazine sulphate, hydroquinone) and acids have been used in this reaction as well as addition of antimony or bismuth (Drummond and Maher 1995; Karthikeyan et al. 2004). The chemistry of phosphomolybdenum blue formation is very complicated and has been recently reviewed comprehensively by Nagul et al. (2015). Additionally, spectrophotometric procedures for phosphate monitoring include other methods. The yellow vanadomolybdate complex method (Gonzales et al. 2016; Munoz et al. 1997; Pons et al. 2006) and the malachite green method (Munoz et al. 1997; O’Toole et al. 2007) are some of the examples.

Batch methods for phosphorus determination involve a number of time- and reagent-consuming steps. By contrast, flow techniques provide accurate, precise and fast phosphorus determination with lower or higher degree of automation depending on the method. The features, advantages and disadvantages, of the most popular flow techniques in phosphorus determination have been reviewed in several papers (Estela and Cerda 2005; Motomizu and Li 2005; Worsfold et al. 2016). Many flow injection analysis (FIA) systems have been reported (Benson et al. 1996; Drummond and Maher 1995; Fiedoruk et al. 2014; Higuchi et al. 1998; Karthikeyan et al. 2004; Kozak et al. 2015; Ruzicka and Hansen 1975). Until now, FIA has been the most popular commercially available flow technique for phosphate determination and monitoring. However, the present day routine assays of orthophosphate are very similar to the oldest design (Ruzicka and Hansen 1975), and the method can be redesigned to be more sensitive and more efficient.

One of the main shortcomings of FIA is its high reagent consumption and incomplete automation. More modern flow systems were optimized and dedicated for phosphate determination: sequential injection analysis (SIA) (Munoz et al. 1997), laboratory on valve (lab on valve (LOV)) (Ruzicka 2000; Wu and Ruzicka 2001), multi-commuted flow injection analysis (MCFIA) (Fernandes and Reis 2002), multi-syringe flow injection analysis (MSFIA) (Almeida et al. 2004) and multi-pumping flow systems (MPFS) (Pons et al. 2006). Usually, most of the aforesaid flow systems adopt commercially available spectrophotometers (Almeida et al. 2004; Drummond and Maher 1995; Higuchi et al. 1998; Kozak et al. 2015; Munoz et al. 1997; Ruzicka 2000; Wu and Ruzicka 2001) which unfortunately are often too complex and versatile for dedicated applications, bulky and too expensive. There is continued interest in developing miniaturized and automated absorption detection devices.

Light-emitting diodes (LEDs) are often employed as a radiation source in many optical measurements replacing a conventional light source (Bui and Hauser 2013; Bui and Hauser 2015; Dasgupta et al. 1993; Macka et al. 2014). They exhibit high brightness, good stability in light intensity, long lifetime, low heat production, low power consumption and low cost. Since LEDs emit a narrow wavelength range, monochromators are not needed, which allows for construction of very simple devices. Commonly LEDs are paired with silicon photodiodes (PD) as detectors and applied in different flow systems dedicated for phosphorus determination (Fernandes and Reis 2002; Gonzales et al. 2016; Karthikeyan et al. 2004). The light intensity can also be detected by a second LED working as a selective photodiode. Pairing two properly selected LEDs, one for emission and one for detection (paired emitter detector diode (PEDD)), enhances selectivity and enables us to prepare a detector dedicated for some specific determinations, where optical filters can be eliminated. Such systems can be employed for analytical purposes, for example, for phosphate determination (Bui and Hauser 2013; Fiedoruk et al. 2014; O’Toole et al. 2007; Saetear et al. 2013; Shin et al. 2016).

In our previous publications and patent, we described a novel type of photometric detector—direct-injection detector (DID) integrated with an MPFS system (Kalinowski and Koronkiewicz 2016; Koronkiewicz and Kalinowski 2011; Koronkiewicz and Kalinowski 2012) which can considerably simplify an analytical flow procedure and allow for reduction in the sample and reagent consumption. In this detector, all the solutions are injected directly into the so-called reaction-detection chamber, in which the reagents are rapidly mixed and the photometric signal is developed. For high precision and fast injection of all the solutions, a system of properly selected solenoid micro-pumps must be applied.

In this work, the photometric DID detector integrated with a MPFS flow system was for the first time optimized and evaluated especially for determination of dissolve reactive phosphorus in environmentally important samples: wastewater as well as surface and groundwater. We intended to construct a novel automated and miniaturized system which allows for very fast phosphorus determination and very low consumption of reagents and energy.

## Materials and methods

### Reagents and solutions

Standard working solutions of phosphate were prepared by appropriate dilution of an AAS-certified reference material (Fluka, Switzerland). Solutions of ammonium molybdate were prepared by dissolving crystalline ammonium molybdate tetrahydrate (Stanlab, Lublin, Poland) in water. Ascorbic acid solutions in water were prepared using a crystalline reagent obtained from Chempur (Piekary Slaskie, Poland). Sulphuric acid, which was used as a carrier, was prepared by appropriate dilution of a concentrated solution obtained from Stanlab (Lublin, Poland). Detergent, sodium dodecyl sulphate (sodium lauryl sulphate (SLS)), was obtained from Aldrich (Steinheim, Germany). The final concentration of the detergent in the carrier solution was less than 0.02%.

Samples of treated wastewater came from the sewage treatment plant in Reszel, Poland. Surface water samples came from Kortowskie Lake and Bukwaldzkie Lake (Olsztyn, Poland). They were filtered immediately after collection through 0.45-μm filters obtained from Macherey-Nagel (Duren, Germany) according to the standard method (Closceri et al. 1998). They were preserved by freezing below − 10 °C. The certified reference groundwater sample ERM-CA616 (European Reference Material, sample no. 0212) was applied without additional pretreatment.

Ascorbic acid solutions were prepared daily; ammonium molybdate and sulphuric acid solutions were prepared weekly. All the solutions were prepared with analytical-grade chemicals and with deionized water obtained from a Milli-Q (Millipore) water purification system (resistivity 18.2 MΩ cm). They were stored in glassware which was cleaned using detergents free of phosphate or in sterile polypropylene disposable vessels.

### Flow system and procedures

**Fig. 1:**
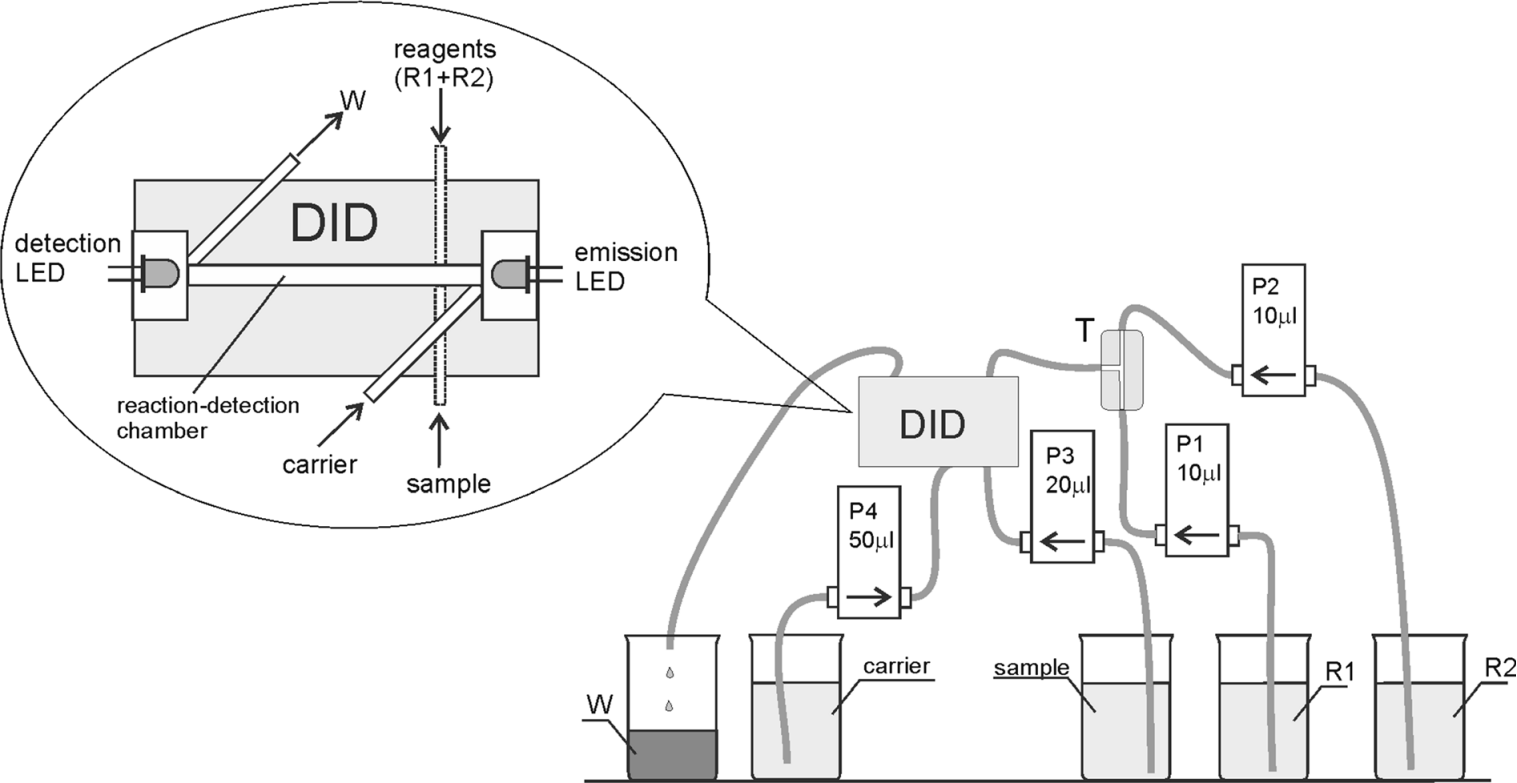
The flow system for orthophosphate determination. DID direct-injection photometric detector, P1, P2, P3, P4 solenoid micro-pumps. T three-way connector, R1 ammonium molybdate, R2 ascorbic acid, W waste

The flow system was designed to employ a direct-injection photometric detector (DID) integrated with four solenoid micro-pumps (Fig. 1). The direct-injection detector was made from one block of Teflon as described in our previous publications (Koronkiewicz and Kalinowski 2011; Koronkiewicz and Kalinowski 2012). Inside this block, there was a tube-shaped reaction-detection chamber of the total volume of about 60 μL. The length of the chamber (i.e. the optical path length) was 20 mm.

The solenoid micro-pumps were responsible for accurate and precise dispensing and transport of all the solution in the flow system. The nominal volume of the pumps was chosen in such a way so as not to exceed the volume of the reaction-detection chamber (60 μL). The solenoid-operated pulse micro-pumps were purchased from Bio-Chem Valve Inc. (Boonton, USA) and have a nominal volume of 10 μL (product no. 120SP1210-4TE) or from Cole Parmer (USA) and have a nominal volume of 20 μL (product no. P/N 73120-10) and 50 μL (product no. P/N 73120-22). The flow lines were made of a PTFE tube (ID 0.8 mm), obtained from Bio-Chem Valve Inc. (Boonton, USA, product no. 008T16-080-20).

Thanks to the solenoid-operated diaphragm micro-pumps, the sample and the reagents were injected rapidly (Fortes et al. 2009). The analytical reaction began at the very moment of injection into the reaction-detection chamber. The construction of the DID detector promoted effective mixing because all the solutions were injected in counter-current, at the same time. The coloured product was created on the optical path of the detector, and this process was monitored at all times by absorbance registration. Two light-emitting diodes, emission and detection LEDs, situated at the opposite sides of the reaction-detection chamber were responsible for absorbance measuring. This way, the process of detection took place “in situ”, at the same place and time as the analytical reaction.

A red, high brightness LED was selected as an emission diode (Kingbright L-7113SEC-H, USA). It was characterized by very intensive light (10,000 mcd) and the maximum emission of 630 nm. The emission spectrum of this LED was matched by the absorption spectrum of the molybdenum blue (Motomizu and Li 2005). The emission LED was powered by a current of 2 mA.

The spectral detection sensitivities of the LEDs are usually as narrow as their emission bands, but shifted to shorter wavelengths compared to the emission maximum (Bui and Hauser 2013; Shin et al. 2016). Therefore, the sensitivity spectra of several LEDs were registered to choose a proper LED as a detector. The system applied for this purpose was similar to the one described in literature (Bui and Hauser 2013; Shin et al. 2016) and contained a tungsten lamp, diffraction grating and the BPW20 photodiode (Vishay, USA) for reference. As the detection diode, a red LED (emission light of *λ*_max_ = 660 nm) purchased at a local electronic parts shop was selected.

The work of the entire system (detector and micro-pumps) was controlled by the electronic equipment developed at our laboratory specifically for flow analysis with photometric detection (Kalinowski, S. Home Page). The LED current was precisely stabilized. The PEDD detector and pulse micro-pumps were PC-controlled. The software was developed in the Delphi programming language and enables us to control the current supplied to the emission LEDs and to record the signal from the detection LEDs. Another task of this system was to control the work of the solenoid micro-pumps and to calibrate the absorbance.

**Fig. 2:**
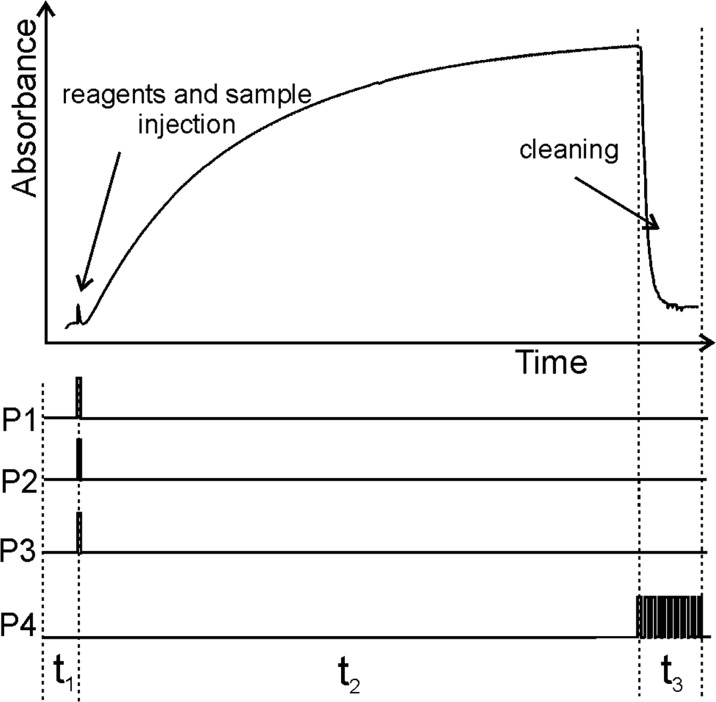
The analytical signal obtained for orthophosphate determination and time diagram of a micro-pumps switching sequence; time for baseline recording (*t*_1_), time for developing and measuring the analytical signal (*t*_2_), time for cleaning the reaction-detection chamber (*t*_3_)

The solenoid pumps were operated individually. The synchronization of all the pumps was possible by appropriate programming of the time switching sequences. The example of the program applied to control the work of solenoid pumps is shown in Fig. 2.

After optimization, the time for baseline recording (*t*_1_) was established equal to about 2 s. The most appropriate time for molybdenum blue creation in stop-flow condition (*t*_2_) was established equal to 20 s. About 8 s were needed to clean the reaction-detection chamber (*t*_3_). As a result, the cycle time equal to 30 s and sample throughput of 120 samples per hour were found.

## Results and discussion

Simple construction and operation of the photometric DID system significantly assist the process of optimization. The commonly required labour-intensive task of instrumental optimization (e.g. sample volume, flow rate, length of the reaction coil, etc.) is practically unnecessary. In principle, only the chemical parameters must be optimized and evaluated. Additionally, application of DID system allows for the coloured product development to be monitored. The kinetic characteristics are often very helpful in the optimizing process.

### Optimization of experimental parameters: reagent concentration

The influence of several physical and chemical parameters was evaluated following the univariate method. The critical parameters for the formation of phosphomolybdenum blue are an appropriate acid, molybdate and reductant concentrations. The high absorbance signal for standard solutions of phosphate and for blank, reaction kinetics and signal repeatability were taken into account. Durability and stability of the reagents over time were also taken into consideration.

Acidity strongly affects the stability of 12-molybdophosphoric acid. It is known that a self-reduction of the molybdate can occur in low acidity (Worsfold et al. 2016), but at higher acidities, sensitivity is quickly lost due to 12-molybdophosphoric acid decomposition into cations (Nagul et al. 2015). The molybdenum blue reaction requires a strong acid, with the pH value generally below 1 to ensure inhibition of direct Mo(VI) reduction. The oxidizing acids (nitric, perchloric) interfere with the reduction process (reaction 2). The majority of described molybdenum blue methods utilize sulphuric acid.

**Fig. 3:**
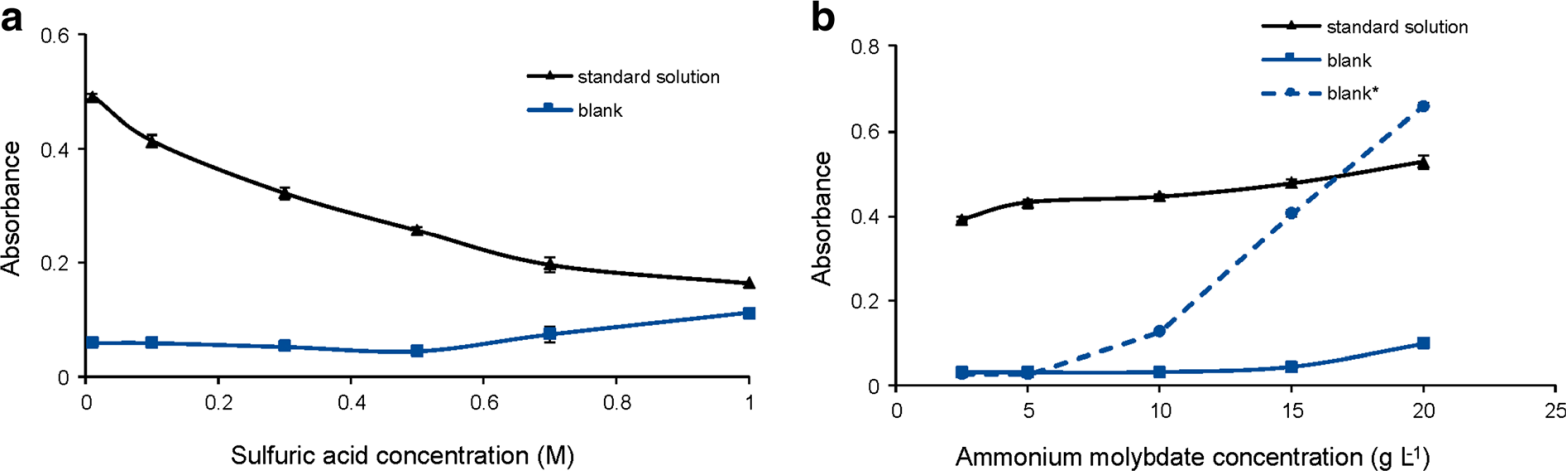
The influence of the sulphuric acid (carrier) (**a**) and ammonium molybdate (**b**) concentrations on the absorbance for the standard solution of phosphate (5 mg L^−1^) and the blank. Ascorbic acid: 60 mmol L^−1^. Absorbance for the blank registered using 5-day-old ammonium molybdate solutions (blank*)

Preliminary investigations indicated that ammonium molybdate solutions prepared in water or in H_2_SO_4_ of concentration lower than 0.5 mol L^−1^ became blue after 1 day of storage at room temperature. Therefore, all ammonium molybdate solutions, due to its stability, were prepared in 0.5 mol L^−1^ H_2_SO_4_. The influence of the sulphuric acid concentration on the analytical signal was examined for carrier solutions. As can be seen in Fig. 3a, with an increase in H_2_SO_4_ concentration, the analytical signal decreased for the standard solution of phosphate, while for blank, the signal increased. Therefore, deionized water was chosen as the carrier, providing the greatest difference between the analytical signal and the blank. Additionally, the carrier solution contained an anionic detergent, sodium dodecyl sulphate (SLS), at the concentration of about 0.05 g L^−1^, which successfully prevented air bubble adsorption inside the reaction-detection chamber of DID, on the optical path.

At the given acid concentration, the amount of 12-molybdophosphoric acid and reduction increases with increasing Mo(VI) concentration. The more Mo(VI) is added to the reaction, the more the formation of 12-molybdophosphoric acid (reaction 1) is favoured. However, excessive amounts of Mo(VI) can result in gains in sensitivity being offset by direct Mo(VI) reduction. This phenomenon is considered to be one of the reasons for high blank in the molybdenum blue method (Nagul et al. 2015). For that reason, it is important to optimize the ammonium molybdate concentration. This concentration was examined in the range of 2.5–20 g L^−1^ (Fig. 3b). A concentration of 10 g L^−1^ was chosen for subsequent experiments as it gives the best signal for the standard solution of phosphate and the blank along with a good repeatability. Unfortunately, at this concentration, it has been observed that the solutions were not stable after about 5 days. For the ammonium molybdate concentration higher than 10 g L^−1^, the increase of blank signal was observed, especially when using solutions that were not fresh.

**Fig. 4:**
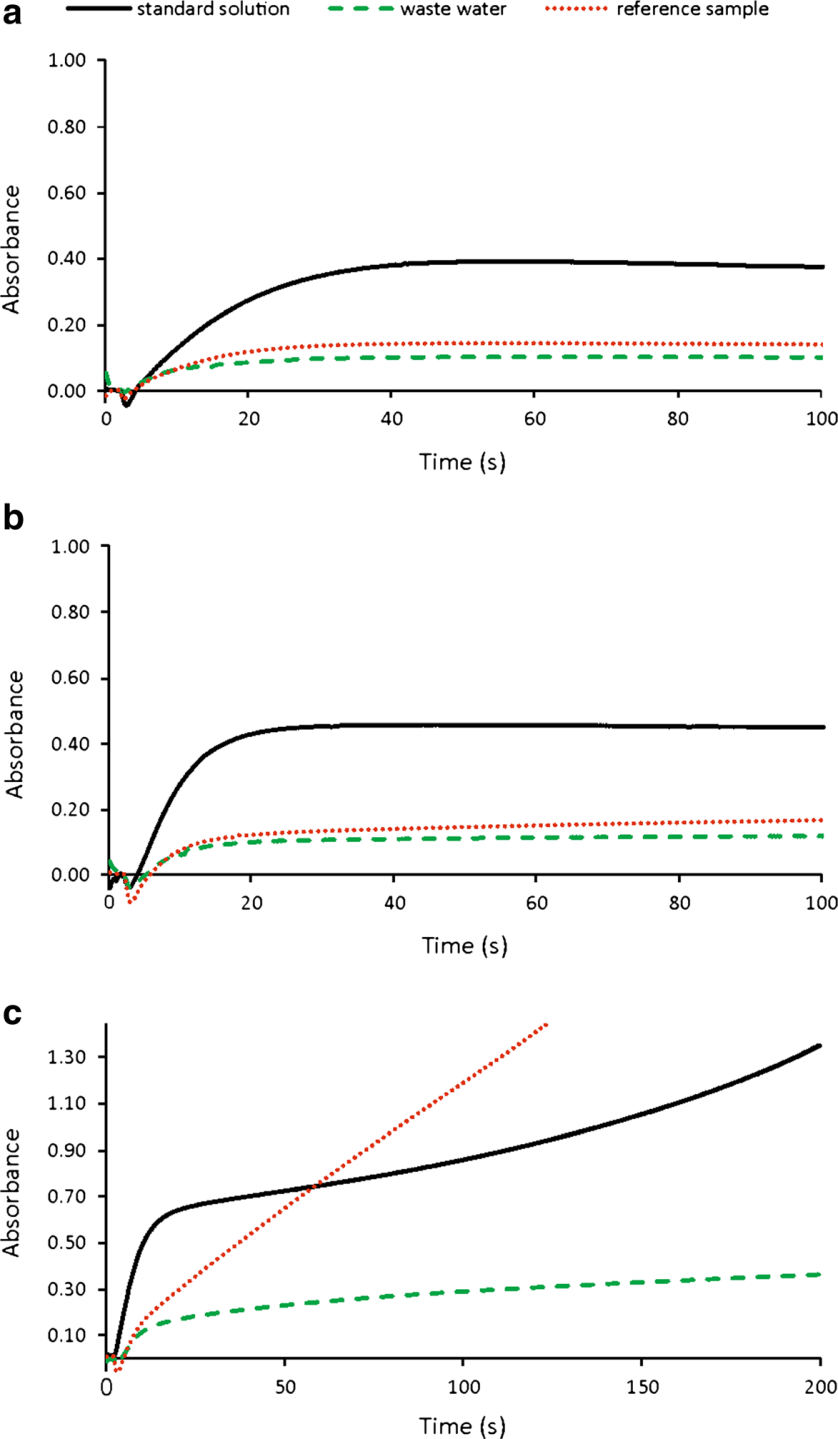
Analytical signals obtained for the standard solution of phosphate (5 mg L^−1^), the wastewater sample and the reference sample. The curves were registered for different ammonium molybdate concentrations. **a** 5 g L^−1^. **b** 10 g L^−1^. **c** 20 g L^−1^

Additionally, it was found that the kinetic characteristics of analytical signals for standard solutions, reference sample and wastewater samples strongly depend on the ammonium molybdate concentration. For the ammonium molybdate solution of 5 and 10 g L^−1^, all the kinetic characteristics were similar (Fig. 4a, b). For higher ammonium molybdate concentrations, the reaction kinetics for all the investigated samples became different. The biggest differences in the shapes of kinetic curves were observed for the concentration of ammonium molybdate of 20 g L^−1^ (Fig. 4c). It was an additional reason to accept the ammonium molybdate concentration of 10 g L^−1^ as optimal. For that concentration, the stop-flow time required to achieve the equilibrium state was equal to about 20 s.

**Fig. 5:**
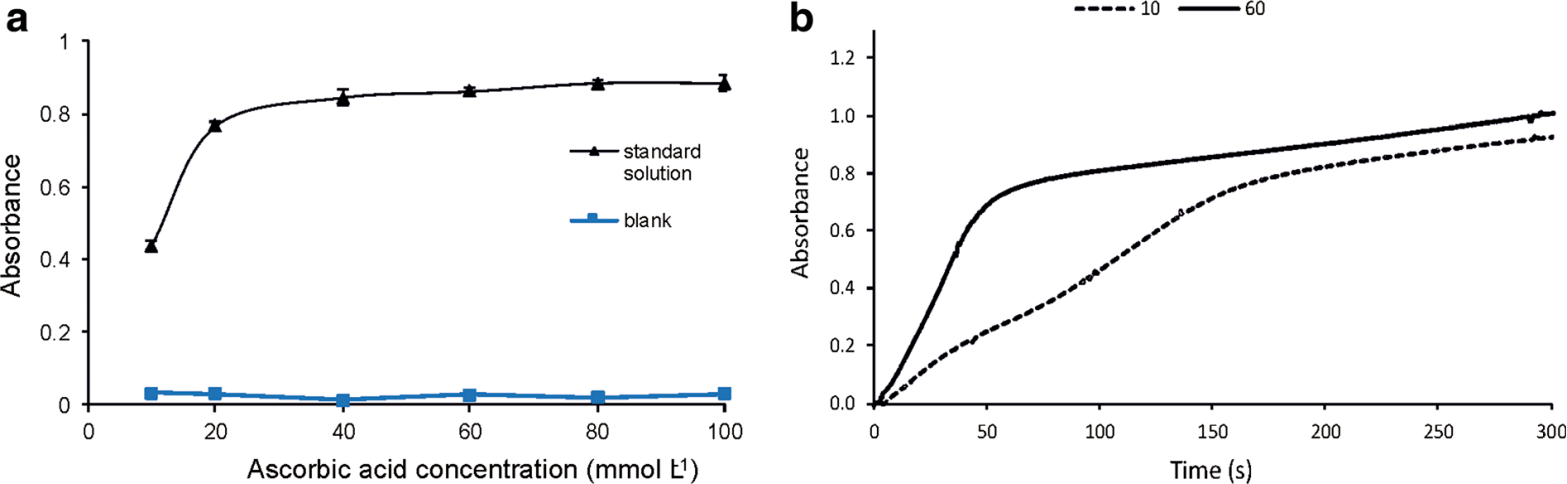
Optimization of ascorbic acid concentration. **a** Influence of ascorbic acid concentration on absorbance for 10 mg L^−1^ standard solution of phosphate and the blank. **b** Kinetic curves for 10 and 60 mmol L^−1^ of ascorbic acid

Although many reducing agents, such as tin(II) chloride, ascorbic acid, hydrazine and hydroquinone, are reported in phosphate analysis, we decided to use ascorbic acid in our experiments. Tin(II) chloride used as a reductant, especially for determination of phosphate in freshwaters, makes the analytical reaction relatively fast, and the absorption coefficient is higher than that of ascorbic acid. However, the tin(II) chloride solution has to be prepared daily because it is unstable (Pons et al. 2006). Therefore, we decided to use ascorbic acid, which is more recommended for monitoring stations because of its stability, high sensitivity and the cost of the analysis. It was previously found that a reagent mixture of ascorbic acid with detergent is stable and can be used for at least 3 months if stored in a refrigerator (Higuchi et al. 1998). Ascorbic acid concentration was optimized to achieve the highest analytical signal and the shortest reaction time. Optimization was performed in the range of 10 to 100 mmol L^−1^. Dependence of the analytical signal and the blank on the ascorbic acid concentration is presented in Fig. 5a. For further experiments, 60 mmol L^−1^ ascorbic acid concentration was chosen as the optimal one. At this concentration, the recommended (Drummond and Maher 1995) large excess of ascorbic acid on the maximum phosphate level was presented.

The excess of ascorbic acid is required to reach the equilibrium of the reaction rapidly. The influence of two ascorbic acid solutions with concentrations of 10 and 60 mmol L^−1^ on the reaction kinetics is shown in Fig. 5b.

### Analytical parameters

The calibration graph for the determination of DRP was found to be linear up to 12 mg L^−1^. The detection limit (LOD), calculated as *3s*_*b*_*/S*, where *s*_*b*_ is the standard deviation for 10 measurements of the blank and *S* is the slope of the calibration graph, is 0.1 mg L^−1^. The working range is sufficient to apply the method for the determination of dissolved orthophosphates in the treated wastewater samples. The repeatability, calculated as a relative standard deviation (RSD) for 10 successive injections of 4 mg L^−1^ standard solution of orthophosphate, was 2.2%. An injection throughput of about 120 injections h^−1^ was achieved (cycle time 30 s.).

The method is characterized by a very low consumption of sample (20 μL) and reagents (10 μL of ammonium molybdate and 10 μL of ascorbic acid) and by a very low volume of generated waste—only 440 μL per analysis.

### Application to the real samples

The accuracy of the proposed method was tested by the analysis of several treated wastewater and lake water samples. The samples were spiked with orthophosphate, according to Table 1. Recoveries in the range of 80 to 106% were observed. The lowest recoveries were achieved for the sample with the lowest level of DRP. The method presents good accuracy considering the complex composition of the samples.

**Table 1:** Results of the determination of DRP in wastewater and lake water samples

No.	Determined concentration of DRP (mg L^−1^)	Added phosphorus standard (mg L^−1^)	DRP found (mg L^−1^)	Recovery (%)
1.^a^	0.43 ± 0.02	2.00	2.41 ± 0.07	99
		4.00	4.55 ± 0.16	103
		6.00	6.55 ± 0.07	102
2.^a^	1.15 ± 0.19	2.00	3.07 ± 0.06	96
		4.00	5.17 ± 0.17	100
		6.00	7.33 ± 0.39	103
3.^a^	0.67 ± 0.02	2.00	2.78 ± 0.05	105
		4.00	4.82 ± 0.07	104
		6.00	7.03 ± 0.23	106
4.^b^	0.26 ± 0.09	2.00	2.25 ± 0.05	99
		4.00	4.31 ± 0.02	101
		6.00	6.45 ± 0.10	103
5.^b^	0.27 ± 0.03	2.00	2.19 ± 0.04	96
		4.00	4.33 ± 0.05	101
		6.00	6.37 ± 0.13	102
6.^c^	Below detection limit	2.00	1.66 ± 0.08	83
		4.00	3.81 ± 0.09	95
		6.00	5.71 ± 0.08	95
7.^c^	Below detection limit	2.00	1.59 ± 0.03	80
		4.00	3.70 ± 0.07	92
		6.00	5.55 ± 0.16	92

Results represent the average of at least four determinations ± SD

^a^Treated wastewater

^b^Lake water

^c^Lake water

The accuracy of the method was also evaluated in the determination of orthophosphate in the certified European Reference Material sample (ERM®-CA616). For assessing the accuracy of the method, the CRM sample was analysed preparing three independent calibration graphs. The results were compared with the certified value as described in the ERM Application Note 1 (European Reference Material’ application note). There was no significant difference between the measured results (2.29 ± 0.13 mg L^−1^) and the certified value (2.24 ± 0.10 mg L^−1^). The results obtained show that the proposed method can be applied to the determination of DRP in samples of complex composition, e.g. lake water and treated wastewater.

### Comparison of proposed system with other flow systems

A comparison of the analytical parameters received with the proposed direct-injection photometric detector integrated with the MPFS system (DID-MPFS) with some of those described previously was carried out. The results are summarized in Table 2. Firstly, the use of a DID system considerably decreases the sample and reagent consumption. For a DID-MPFS system, both the reagent consumption and the total waste are at least one order of magnitude lower than for other methods (microlitre range). Similar volume of sample (20 μL) was used only in lab-on-valve technique. Furthermore, the DID-MPFS system is characterized by the highest throughput equal 120 injections per hour. Usually, the slow kinetics of the molybdenum blue creation is accelerated by increasing the temperature or by introducing a catalyst such as antimony or bismuth (Wu and Ruzicka 2001; Karthikeyan et al. 2004). The use of DID-MPFS system makes it possible to achieve short analysis time without this. After modification of the molybdenum blue reaction chemistry, achieving a throughput higher than 120 injections per hour should not be a problem.

**Table 2:** Analytical features of orthophosphate determination in various flow systems with photometric detection

Flow technique	Detection system	Chemical reaction	Optical path length (mm)	Sample consumption (μL)	Total waste ^a^ (μL)	Repeatability RSD (%)	Working range (mg L^−1^)	Sample throughput (injections h^−1^)	Sample	Ref.
FIA	LED-PD	Molybdenum blue with bismuth	30	200	–	< 5	0.05–4	50	Natural water	Karthikeyan et al. (2004)
FIA	Bimodal LED-LED	Molybdenum blue	5	250	–	–	11.3–300	–	Softdrinks	Fiedoruk et al. (2014)
SIA	LED-LED	Calcium phosphate formation (turbidimetric detection)	10	2250	12,700	2	50–200	21	Cola drinks	Saetear et al. (2013)
LOV	Tungsten-halogen lamp, spectrophotometer, fiber-optic cable	Molybdenum blue with antimony tartrate	5	20	–	0.80	0.001–0.030	60	Lake water and tap water	Wu and Ruzicka (2001)
MPFS	Spectrophotometer	Vanadomolybdate	10	1300	3900	0.6	0.08–20	75	Wastewater	Pons et al. (2006)
MPFS	LED-PD	Vanadomolybdate	10	400	2800	2.4	0.95–50	36	Sewage and wastewater	O’Toole et al. (2007)
MPFS	DID LED-LED	Molybdenum blue	20	20	440	2.2	0.1–12	120	Wastewater and freshwater	This work

*RSD* relative standard deviation for 10 independent analyses of sample

^a^Total waste (μL/determination)

The repeatability for all the compared systems was below 2.5% and typical for automated or semiautomated methods. Working range depended strongly on the analytical reaction involved in the detection process and on the optical path length. In our system, the optical path length was equal to 20 mm, and we did not use catalysts. The working range was fitted to the concentration of orthophosphate in wastewater and freshwater samples. If it is necessary to lower the detection limit, it is possible to apply the reaction-detection chamber with longer optical path and/or add antimony tartrate to ammonium molybdate reagent.

And finally, some of the presented flow techniques were combined with expensive spectrophotometers. This excludes miniaturization of the entire measurement system. LED-based technology, especially when dedicated to one type of determination, allows for radical simplification of detection system which is inexpensive and more reliable.

## Conclusions

A novel automatic, low-cost and environmentally friendly method dedicated for determination of the most bioavailable form of phosphorus in aquatic ecosystems—DRP—has been developed and presented for the first time. The proposed method allows for obtaining the better analytical parameters with a more ecological, cheap and reliable technical construction of the flow system than previously described. The main advantages of the method are the low reagent consumption (only 440 μL of total waste per analysis) and high sampling frequency (120 samples per hour), good accuracy and high precision. The method is characterized by sufficient sensitivity and working range for determination of DRP in wastewater treatment plants. Therefore, the approach presented in this publication has a chance to find many practical applications.
